# Differences between professionals’ views on patient safety culture in long-term and acute care? A cross-sectional study

**DOI:** 10.1108/LHS-11-2020-0096

**Published:** 2021-09-08

**Authors:** Mari Liukka, Markku Hupli, Hannele Turunen

**Affiliations:** Department of Nursing Science, University of Eastern Finland, Kuopio, Finland and South Karelia Social and Health Care District, Lappeenranta, Finland; Department of Rehabilitation, South Karelia Social and Health Care District, Lappeenranta, Finland; Department of Nursing Science, University of Eastern Finland, Kuopio, Finland and Kuopio University Hospital, Kuopio, Finland

**Keywords:** Patient safety, Patient safety culture, Management, Long-term care, Acute care, Professional

## Abstract

**Purpose:**

This paper aims to assess how patient safety culture and incident reporting differs across different professional groups and between long-term and acute care. The Hospital Survey On Patient Safety Culture (HSPOSC) questionnaire was used to assess patient safety culture. Data from the organizations’ incident reporting system was also used to determine the number of reported patient safety incidents.

**Design/methodology/approach:**

Patient safety culture is part of the organizational culture and is associated for example to rate of pressure ulcers, hospital-acquired infections and falls. Managers in health-care organizations have the important and challenging responsibility of promoting patient safety culture. Managers generally think that patient safety culture is better than it is.

**Findings:**

Based on statistical analysis, acute care professionals’ views were significantly positive in 8 out of 12 composites. Managers assessed patient safety culture at a higher level than other professional groups. There were statistically significant differences (*p* = 0.021) in frequency of events reported between professional groups and between long-term and acute care (*p* = 0.050). Staff felt they did not get enough feedback about reported incidents.

**Originality/value:**

The study reveals differences in safety culture between acute care and long-term care settings, and between professionals and managers. The staff felt that they did not get enough feedback about reported incidents. In the future, education should take these factors into consideration.

## Introduction

Patient safety (PS) is an important concern for health-care organizations where both managers and health-care professionals have the important and challenging responsibility of promoting it. For managers to achieve this goal, they are tasked with clearly communicating safety priorities to staff and implementing PS procedures (Mattson *et al.*, 2015). A study by Auer *et al.* (2014) reported that only half of the nursing staff felt that the actions of managers show that PS is their number one priority. Thus, if managers do not constantly communicate about safety, the staff might consider that the organization is not sufficiently interested in incidents or errors (Mattson *et al.*, 2015).

Patient safety culture (PSC) is part of the organizational culture and is associated for example to rate of pressure ulcers, hospital-acquired infections and falls (Braithwaite *et al.*, 2016). Creating a safety culture can be described as a circle that begins with managers defining clear PS targets with their staff. This action will improve the safety practices in a unit and lead to the manager setting additional, more ambitious PS targets (Phipps and Ashcroft, 2014). Managers must be competent at coaching the staff to innovate and create new ways to work and achieve the PS goals. Proficient managers can motivate and empower staff to constantly achieve better results in PS outcomes (Doody and Doody, 2012).

Incident reports are crucial for safety management. By analyzing these reports, managers can identify strategies to prevent future incidents (Qin *et al.*, 2015). Several studies have found that nurses report PS incidents more often than physicians (AbuAlRub *et al.*, 2015; Bagenal *et al.*, 2016), but physicians report severe incidents – such as patient deaths – more often than other health-care professionals (Howell *et al.*, 2015). Another study has provided evidence that health-care professionals consider incident reporting worthwhile and part of their job (Brasaite *et al.*, 2015). They think incident reporting has positive effects on patient safety culture Wami *et al.* (2016), and it is the main instrument to communicate about PS issues. This characteristic of health-care professionals will positively affect PS and may serve as an indicator of the staffs’ attitude toward safety and team culture (Anderson *et al.*, 2013).

However, encouraging staff to participate in analyses of incidents and open discussions about PS problems seem to be difficult (Anderson *et al.*, 2013; Alqubaisi *et al.*, 2016; Macrae, 2016). Nevertheless, this endeavor is necessary for better PS and PSC. A manager must – and some already do – collaborate with staff to communicate openly and in a fair blame manner about PS issues and have meetings about this topic even if an adverse event had not just happened. Communication with the whole team gives a broader view about adverse events (Barros *et al.*, 2014; Correia *et al.*, 2017; Liukka *et al.*, 2018). Staff often feel that they do not have sufficient time for these types of activities, and it has also been suggested that they will wait for somebody to fix the problem after they have reported the incident (Anderson *et al.*, 2013; Alqubaisi *et al.*, 2016; Macrae, 2016). Staff should understand that they are also responsible for their own practice, and open communication about adverse events is necessary so that they can learn from them. They also have to support each other when adverse events occur [World Health Organization (WHO), 2015].

Barriers to reporting incidents include a lack of feedback about reports, ineffective communication to changes in reporting practices, the perception that reporting an event will not help the patient, and fear of blame or punishment and/or the patient making a claim (AbuAlRub *et al.*, 2015; Bagenal *et al.*, 2016; Farokhzadian *et al.*, 2018; Gandhi *et al.*, 2018; Hamdan and Saleem, 2018; Howell *et al.*, 2015; Qin *et al.*, 2015). Unfortunately, managers’ actions do not usually involve enough feedback about PS reports, and the action of giving feedback alone is not sufficient for improving PS (Anderson *et al.*, 2013; Auer *et al.*, 2014; Kvist *et al.*, 2013). Managers generally think that PSC, including open communication and communication about errors, is better than it actually is in practice (Danielsson *et al.*, 2019; Hickner *et al.*, 2016; Richter *et al.*, 2015; Turunen *et al.*, 2013; Willmott and Mould, 2018).

A lack of commitment, non-supportive management, and non-participative decision-making are managers’ actions that will impede improvements in safety culture (Farokhzadian *et al.*, 2018). On the other hand, interactive and visionary leadership styles – such as transformational leadership – are positively correlated with incident reporting, PS outcomes, the staff’s organizational commitment and stronger PSC. Furthermore, transformational leadership styles have led to impressive reductions in medication errors and patient mortality (Al-Yami *et al.*, 2018; Braithwaite *et al.*, 2016; Hillen *et al.*, 2017; Wong *et al.*, 2013).

There are many studies about patient safety culture in acute care but fewer in long-term care. While there are discouraging reports about the poor PSC in long term health-care services, there is much less information about long-term care (The Mid Staffordshire NHS Foundation Trust, 2013). Similar issues have appeared in the public discussion in Finland, where long-term care facilities have been investigated and problems in the quality of care have been observed in the past two years. Thus, it is important to measure PSC both from long-term care and acute care perspectives.

In 2010, a full-time patient safety coordinator started working in the study organization and the organization began using voluntary incident reporting system in January 2010. During the period 2010 to 2016, many trainings about patient safety issues, incident reporting, and analysis were carried out focusing on a non-punitive, blame-free culture.

The following research questions were addressed in this study:

RQ1. How do PSC perceptions differ between managers and other professionals?

RQ2. How do PSC perceptions differ between long-term and acute care?

## Method

### Questionnaire

We used the Hospital Survey on Patient Safety Culture (HSPOSC) 1.0 questionnaire developed by the Agency for Healthcare Research and Quality (AHRQ) to assess the safety culture at the studied health-care organization. AHRQ has also developed Nursing Home Survey on Patient Safety Culture. That questionnaire has been designed specifically for nursing homes. Both questionnaires contain 12 composites – with three to four items each – which measure specific areas of PSC. Items are graded on a five-point Likert type scale (1 = strongly disagree/never, 2 = disagree/rarely, 3 = neither/sometimes, 4 = agree/most of the time or 5 = strongly agree/always). The HSOPSC questionnaire also includes two items that are outside of the composites: *Patient safety grade* and *Number of event reported* (The Mid Staffordshire NHS Foundation Trust, 2013). The Nursing Home Survey also includes two items that are outside of the composites: *I would tell friends that this is a safe nursing home for their family* and *Please give this nursing home an overall rating on resident safety*. Respondents grade the item *Patient safety* using a five-point Likert type scale (1 = excellent, 2 = very good, 3 = acceptable, 4 = poor or 5 = failing). However, the scale was reversed – i.e. a score of 1 signifies failing, while 5 signifies excellent – during the data analysis phase. In analyzing the scale, it was shifted so that 1 means failing and 5 means excellent. This change made the results easier to understand because lower scores reflected more negative results from the PSC perspective. In addition, the questionnaire included a section on background information, which covered occupation, gender, working years in the profession, working years in the hospital, and working years in the current unit. We also added a question to determine if participants get to know whether there are any changes made based on incident reports.

### Data collection

The data were collected in spring 2016. This study was carried out in long-term care (wards and nursing homes) and acute hospital at one integrated Finnish health-care organization. The organization has one central hospital with 277 beds in 2015, nursing homes and long-term wards with 888 beds. In this organization, registered nurses and practical nurses are the biggest professional groups (65% of 4,600 staff members). Education in the vocational college for practical nurses is 180 credits, targeting at level 4 of learning outcomes on the European Qualification Framework (EQF), while registered nurses’ education is 210 credits targeting at level 6 on EQF learning outcomes [European Union (EU), 2018].

We also used data from the organizations’ incident reporting system to determine the number of reported PS incidents. The study organization had started using it since 2010; all health-care professionals can anonymously report PS incidents via this electronic reporting system.

### Ethical considerations

Ethical approval for the study was granted by the University Committee on Research Ethics. Permission to perform the study was also obtained from the study hospital. Participants were informed about the voluntary and anonymous nature of their participation in a cover letter that described the intended research and contact information of the researchers.

### Sampling

The researchers sent an e-mail with a link to the questionnaire using the organization’s ready-made e-mail lists for hospital staff. For the units without ready-made lists, the researchers sent an e-mail to managers in which they asked the managers to forward the link to their staff. After two weeks, employees were sent a reminder to answer the questionnaire. The link was sent to 1,404 hospital staff members. After processing all of the completed questionnaires, 374 questionnaires were included in the study, translating to a response rate of 27%.

### Data analysis

Data were analyzed with SPSS for Windows (version 24.0, IBM Corporation, Armonk, NY), with the threshold for statistical significance set at *p* < 0.05. Frequencies and percentages were calculated for all of the items. Composite variables were analyzed and compared as means. The normal distribution of composite variables was tested using the Kolmogorov–Smirnov test; the tests revealed non-normal distributions. The Kruskal–Wallis test was used to compare differences between professional groups and the Mann–Whitney U test was used to compare differences between working areas. Cronbach’s alpha (Table 1) ranged from 0.55 (*Handoffs and transition*) to 0.80 (*Feedback and communication about error)*.

We also calculated the positive response rate (PRR) to PSC composites. Specifically, scores 4 and 5 are positive, 3 neutral, and 1 and 2 are negative responses to an item. PS items with a PRR of at least 75% could be considered strong, whereas items with a PRR lower than 50% require improvement (Sora *et al.*, 2016). Only PRRs are reported.

## Findings

Participants’ background characteristics are described in Table 2. Most of the participants were female (95.1%) and either staff nurses (36.8%) or practical nurses (43.6%). There was a clear minority of physicians; they only comprised 5.1% of participants, and all of them were working in acute care. More than half of the participants (54.3%) worked in long-term care, including wards and nursing homes; the rest (46.5%) worked in an acute hospital. With regard to long-term care, 76% of participants were practical nurses. Many participants had worked in their current unit for 1 to 5 years (36.9%) but had been in their current occupation for 21 years or more (38.6%).

Table 3 shows the PRRs of 12 PSC composites by different professionals and by health-care organizations. The following PSC composites showed the most positive responses: *Handoffs and Transitions* (78.9%) and *Teamwork across units* (75.1%). *Manager expectations and actions promoting patient safety* (51.3%) and *Management support for patient safety* (51.6%) had the fewest positive responses. Managers’ perceptions were most positive in 7 out of the 12 PSC composites among professionals; there was even a 100% PRR in *Communication openness*. Furthermore, managers had over 90% PRRs for PSC composites *Feedback and communication about errors* (91.3%) and *Handoffs and Transitions* (91.3%). Other professional groups did not reach over a 90% PRR in any PSC composite. Practical nurses’ answers were alarmingly low (under 50%) in two composites: *Management support for patient safety* and *Nonpunitive response to errors*.

Based on statistical analysis (Mann–Whitney U test), acute care professionals’ views were significantly positive in 8 out of 12 composites (Table 4). Only *Frequency of events reported* was significantly positive from the long-term care point of view. The *Nonpunitive response to error* mean was below 3.00 in both acute and long-term care. *Staffing* also scored below 3.00 in long-term care.

### Incident reporting, learning and giving feedback

**Figure 1.**
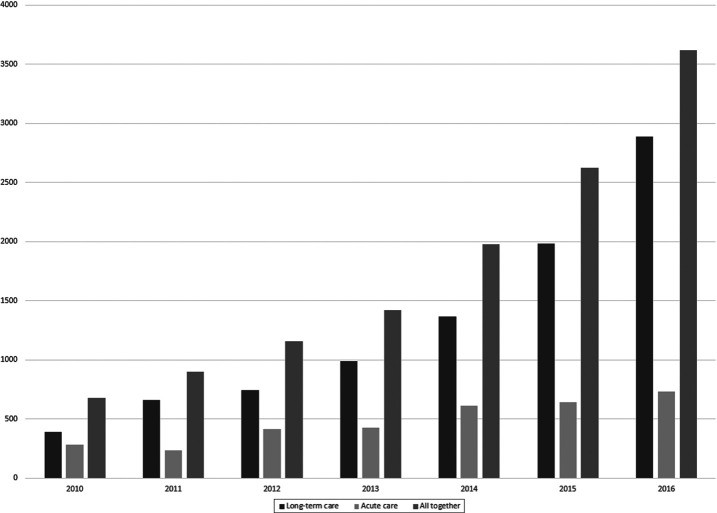
Number of incident reports

In the study health-care settings, the professionals had made 3,755 incident reports in 2016, including 679 (18.1%) near misses and 3,076 (819 %) adverse events. Many of the incident reports (75%) were made in long-term care. Most of the reported incidents were medical related (*n* = 1263, 31.3%), followed by accidents such as falls (*n* = 1068, 28.4%) and communication-related issues (*n* = 360, 8.9%). Of all reported accidents, only 12 occurred in acute care. A high rate of falls explains why there are so many adverse events compared to near misses. The number of incident reports generated by the organization has increased every year since the introduction of the reporting system in 2010. In the beginning, the long-term care staff made 382 reports and acute care staff made 286 (Figure 1). During this period, most reported categories were “medication related” and “communication related” in acute care and “accidents” and “medication related” in long-term care.

Almost every tenth (*n* = 267 or 7%) of the incident reports were in the phase of waiting for manager’s processing. The outcome of those incident reports that were already processed by the manager was in 66% cases to “develop a plan” to ensure that such incidents would not happen again.

There was a difference between frequency of incidents reported and professional groups. Practical nurses made over half (51%) and registered nurses made 38% of incident reports. Every hundredth report (1%) was made by a physician. Information as to whether the reporter is a manager is not available in reporting system. The participants were asked how many times during the past 12 month they had reported PS incidents in the hospital reporting system. Overall, 1–5 reports were made by 65% practical nurses, 63.5% of registered nurses and 52.5% of managers; 6–20 reports were made by 13.5% of practical nurses, 17.5% of registered nurses and 26% of managers. Almost half of physicians (47.4%) responded that they had not reported at all in the past 12 months; the rest of them reported one to five times. There were statistically significant differences (*p* = 0.021) in *Frequency of events reported* between professional groups and between long-term and acute care (*p* = 0.050).

Managers were mostly of the opinion (mean 3.86) that the organization had learned from mistakes that had led to positive changes when comparing results to all other professionals (*p* = 0.000). There was also a statistical significant difference in giving feedback and communication about errors, including changes made based on incident reports, information about errors that happened in one’s own unit, and discussions to prevent adverse events (*p* = 0.000). Managers’ views were most positive (mean 4.12), whereas practical nurses views were most negative (mean 3.18). Practical nurses estimated this composite more negatively compared to managers (*p* = 0.000) and registered nurses (*p* = 0.011). Further, practical nurses had the lowest mean and number of PRRs compared to the other professional groups.

### Management and patient safety culture

The mean score for *Manager expectations and actions promoting patient safety* was 3.55. Managers (*n* = 23) scored the items *My manager seriously considers staff suggestions for improving patient safety* (mean 4.26) and *Whenever pressure builds up, my manager wants us to work faster, even if it means taking shortcuts* (mean 4.39) better than practical nurses (*n* = 163, means 3.48 and 3.51, respectively, *p* = 0.015) and registered nurses (*n* = 137, means 3.58 and 3.67, respectively, *p* = 0.043). There were also significant differences (*p* = 0.013) among all professional groups in the item *My manager overlooks patient safety problems that happen over and over*.

Managers estimated *Hospital management provides a work climate that promotes patient safety* higher (mean 3.91) than practical nurses (mean 3.21, *p* = 0.032). They also scored *The action of hospital management show that patient safety is a top priority* at a statistically higher level (mean 3.78) compared to practical nurses (mean 3.19, *p* = 0.032).

## Discussion and recommendations

### Limitations and strengths of the study

The HSOPSC scale is designed for the hospital environment and is widely used on the international level (Agency for Healthcare Research and Quality, 2016). The scale has been translated from English into Finnish. The Finnish version of the scale has been used in previous studies with sufficient levels of validity and reliability (Kvist *et al.*, 2013; Turunen *et al.*, 2013). Therefore, a pilot test prior to the use of the questionnaire was unnecessary. Cronbach’s alpha values for four of the PSC composites were acceptable (>0.70). Other composites give lower values but are at same level with previous studies (Waterson *et al.*, 2019).

In this study, long-term care represents wards in health centers and nursing homes in elderly care. Half of the participants were working in nursing homes where staff is available all the time. The HSOPSC is used in this study for all participants, even if there is a developed Nursing Home Survey on Patient Safety Culture (AHRQ, 2020). Use of HSOPSC may have affected the results, although the language of the survey was generalized to respond to different kinds of working areas and the organization’s own terms.

The amount of data differed among the professional groups so there are limitations of reliability when comparing professional groups. Managers principally represent nursing managers, so they can be thought to represent the nursing management of the study organization. The smallest group was “other” (*n* = 4). Due to the low number of answers, the numbers presented in the tables were not discussed in the text, even though there was a very low PRR in some composites. The response rate was 27%. In the study organization, the biggest health-care professional groups are registered nurses, practical nurses and physicians. A higher response rate may have increased the number of physicians among the participants, but this is not likely to influence the study results significantly.

Two different kinds of statistical methods have been used to compare working units’ PSC – the Mann–Whitney U-test and the rate of positive responses. In some composites, these methods gave contradictory results, but overall, acute care professionals scored better on several composites with both methods than long-term care professionals. The reason for this contradiction might be that positive responses included only 4 = “agree” and 5 = “strongly agree” from the scale, but for the Mann–Whitney U-test, the whole scale from 1 to 5 was included.

### Discussion about study results

*Handoffs and transition* was the most positively scored composite. The study organization has a common electronic patient record system, a factor that can explain this result. Professionals are able to see written information about a patient no matter where they work in the organization. Data is transferred automatically and it is not related to professionals’ memory.

The worst results were in composites that are related to managers’ expectations and actions to promote and support PS. The staff felt that the organization is neither learning from the reported mistakes nor providing enough feedback. This approach does not support experiential learning, and the lack of feedback may cause staff to think that managers have little to no interest about reporting incidents (Auer *et al.*, 2014; Mattson *et al.*, 2015). Second, the lack of feedback and communication about incident reports may indicate that staff do not know the process involved after report has been made. As has been suggested in the literature, a manager’s lack of attention regarding their perception by staff may decrease the number of reports and hurt the organization’s PSC (Anderson *et al.*, 2013; Correia *et al.*, 2017; Farokhzadian *et al.*, 2018; Gandhi *et al.*, 2018; Mattson *et al.*, 2015). This study showed that managers themselves know that there is lot of work to do with regard to learning from adverse events.

An organization’s incident reports support staffs’ opinion about organizational learning and has positive effects on PSC (Wami *et al.*, 2016). This study was carried out during the spring and the number of incidents is referring to that of the whole year of 2016, so there is a possibility that participants were activated to report more often about patient safety incidents. While Figure 1 shows an increasing trend in total number of incidents reported from 2010 to 2016, the growth is less in acute care compared to long-term care. The absolute number of incidents reported annually is also considered low. A relatively small number of reports will certainly still lead to action in developing specific plans to address issues; however, this may lead to a decreased number of incident reports. Based on previous study, lack of feedback and the feeling that reporting is not worth it are the main barriers to reporting patient safety incidents (AbuAlRub *et al.*, 2015). Decreasing numbers of incident reports make managers’ work to promote patient safety more challenging.

One alarming finding is that long-term care professionals reported more incidents compared to acute care but their feelings about *Organizational learning* and *Feedback and communication about error* were at lower levels. Half of all reported incidents were accidents. The most common accidents were falls. It could be presumed that falls are easier to report since professionals are not usually held responsible for a patient’s fall, if the patient falls alone. This seems to be the main reason for a large number of reported incidents, although measured PSC is at a low level. Long-term care professionals even scored *Nonpunitive response to errors* lower, although acute care also scored that composite below 3.00. These data signal that PSC is not at a good level. Managers may not have a good attitude with regard to PS or their action to promote PS is not visible to professionals.

The studied organization offered ample incident reporting and analysis training. The education – which focused on feedback and communication on errors – was voluntary, and it is well-known that nursing staff participated more than physicians. This might be one reason why physicians report patient safety incidents less often than nursing staff, although physicians’ low reporting activity has been previously observed (Howell *et al.*, 2015). The development of incident reporting must happen in units, and managers are responsible for this implementation. Even if managers are responsible for arranging team meetings about incident reports and PS topics, staff should also be active and ask about these meetings. If they hear nothing about incident reports that have been made in their unit, they should ask about those. One problem – which has also been previously highlighted – is that education at the organizational level is often quite general; as such, participants have a passive role, and there is not enough time to focus on each unit’s PS problems (Farokhzadian *et al.*, 2018).

The analyses revealed that managers graded several composites higher than other professional groups. This outcome supports previous findings that managers estimate PSC more positively, when compared to other professionals (Danielsson *et al.*, 2019; Hickner *et al.*, 2016; Richter *et al.*, 2015; Turunen *et al.*, 2013; Willmott and Mould, 2018). One important point is that practical nurses took the most negative attitude toward management and patient safety. They felt that they did not get not enough feedback about reported incidents and they do not know if the reports have led to any changes in action. Notably, most incident reports were made in long-term care, the setting that contained the majority of the practical nurses who participated in this study. Previous research has suggested that health-care managers’ responsibilities include giving feedback about PS incidents, talking more openly about the incidents that occur in units, and working on new strategies for preventing similar incidents in the future (Qin *et al.*, 2015). Based on this study’s results, in future, managers’ negligence toward PS could be seen in the number of reported incidents.

### Recommendations

There has been substantial work toward better PS and PSC over the years. Nevertheless, it seems that there is still a lot of work to be done. This current results are in line with previous studies Anderson *et al.* (2013), Alqubaisi *et al.* (2016) and Macrae (2016) about managers’ skills to empower staff to participate in incident analysis and open discussion about PS. A system for better communication between organizational management and professionals should be created, and the goals of PS action also have to be clear to staff (Mattson *et al.*, 2015; Phipps and Ashcroft, 2014).

Physicians’ low number of incident reports is also in line with previous findings (Howell *et al.*, 2015). Organizations should involve patient safety education in a mandatory introduction to work for all health-care professionals. The training should include basic information about incident reporting: When do you report? How do you report? Whose responsibility is it to report? Discussion about a fair-blame and non-punishing culture, as well as the theory about why errors happen, should also be carried out to improve the PSC.

Mangers have a key role in creating better PSC. They definitely need education about PS, incident reporting and analyzing incident reporting. Based on this study’s results, it should be interesting to study the differences between long-term care and acute care managers’ education, management style and experience. More PSC studies in the long-term care environment are also needed.

## Conclusion

While the provision of feedback and discussing errors have both been emphasized as crucial components of strong PSC, the staff at the studied hospital still felt that they are not well informed about the errors that occur in their units. This deficit may lead to situations in which staff do not report errors because they feel that reports are not important. The present study indicates that future education on incident reporting and analysis must take individual units into account.

Based on this study the acute care professionals have more positive views about PSC than long-term care professionals. The development of an organizational culture takes time. Specifically, it takes time for managers to get to know their staff and find ways to motivate them to work toward the required PS level. However, it is important to remember that the manager’s actions will have a marked influence on whether the unit will reach the set goals.

## Figures and Tables

**Table 1.** Internal consistency evaluated with Cronbach’s *α*

Patient safety culture dimension	Cronbach’s *α*
Teamwork within units	0.76
Manager expectations and actions	0.78
Organizational learning	0.62
Management support for patient safety	0.75
Overall perceptions of patient safety	0.61
Feedback and communication about error	0.80
Communication openness	0.63
Frequency of events reported	0.68
Teamwork across units	0.57
Staffing	0.60
Handoffs and transitions	0.55
Nonpunitive response to errors	0.69

**Table 2.** Background characteristics

	Long-term care		Acute care		Total	
	N	(%)	n	(%)	n	(%)
*Gender*						
Female	193	96.5	153	87.9	346	95.1
Male	3	1.5	15	8.6	18	4.9
Total	196	53.8	168	46.2	364	100
*Occupation*						
Manager	7	3.5	16	9.2	23	6.2
Registered nurse	37	18.5	100	57.5	137	36.8
Practical nurse	152	76	11	6.3	163	43.8
Physician	0	0	19	10.9	19	5.1
Investigation and rehabilitation staff	0	0	26	14.9	26	7
Other	3	1.5	1	0.6	4	1.1
Total	199	53.5	173	46.5	372	100
*Working years in current unit*						
Less than 1 year	28	14	15	8.6	43	11.7
1 to 5 years	99	49.5	39	22.4	138	37.5
6 to 10 years	44	22	45	25.9	89	24.2
11 to 15 years	7	3.5	24	13.8	31	8.4
16 to 20 years	10	5	18	10.3	28	7.6
21 years or more	12	6	27	15.5	39	10.6
Total	200	54.3	168	45.7	368	100
*Working years in current occupation*						
Less than 1 year	7	3.5	4	2.3	11	3
1 to 5 years	34	17	17	9.8	51	14
6 to 10 years	42	21	34	19.5	76	20.8
11 to 15 years	25	12.5	29	16.7	54	14.8
16 to 20 years	35	17.5	31	17.8	66	18.1
21 years or more	50	25	57	32.8	107	29.3
Total	193	52.9	172	47.1	365	100

**Table 3.** Percentage of positive response rates*^)^ in professional groups and in working area

Composite	Manager	Registered nurse	Practical nurse	Physician	Investigation and rehabilitation staff	Long-term care	Acute care	Positive responses of all sample
Teamwork with in units	39.1	59.1	54.0	31.6	34.6	57.0	47.7	52.7
Management expectations and actions promoting patient safety	39.1	52.6	53.4	63.2	38.5	54.5	47.7	51.3
Organizational learning – continuous improvement	78.3	62.0	56.4	68.4	80.8	55.5	69.0	61.8
Management support for patient safety	82.6	46.0	49.7	47.4	69.2	50.5	52.9	51.6
Overall perceptions of patient safety	65.2	67.2	71.2	63.2	53.8	72.0	62.6	67.6
Feedback and communication about error	91.3	70.1	54.0	63.2	76.9	56.0	74.7	64.7
Communication openness	100.0	73.7	63.2	84.2	73.1	65.0	78.2	71.1
Frequency of events reported	87.0	62.0	72.4	42.1	53.8	73.5	58.6	66.6
Teamwork across units	69.6	75.9	*77.9*	73.7	65.4	77.0	79.3	75.1
Staffing	69.6	59.1	65.6	68.4	69.2	64.5	62.6	63.6
Handoffs and transitions	91.3	76.6	76.7	84.2	88.5	78.5	79.3	78.9
Nonpunitive response to errors	87.0	56.2	44.8	73.7	69.2	49.0	62.6	55.3
Mean	75.0	63.4	61.6	63.6	64.4	62.8	64.6	63.4

Note: *^)^ Agree and strongly agree, negative worded questions have been recoded

**Table 4.** Differences*) in patient safety culture composites between long-term and acute care

Composite	Long-term care			Acute care			
	N	Mean	SD	N	Mean	SD	*p*-value*
Teamwork within units	193	3.55	0.676	171	3.82	0.633	0.000
Manager expectations and actions	197	3.46	0.846	173	3.66	0.819	0.015
Organizational learning	192	3.28	0.646	170	3.49	0.642	0.001
Management support for patient safety	197	3.18	0.813	167	3.27	0.860	0.332
Overall perceptions of patient safety	194	3.20	0.679	168	3.34	0.739	0.042
Feedback and communication about error	199	3.23	0.907	171	3.63	0.752	0.000
Communication openness	197	3.40	0.746	174	3.73	0.669	0.000
Frequency of events reported	198	3.63	0.760	169	3.47	0.821	0.050
Teamwork across units	197	3.38	0.549	171	3.34	0.595	0.767
Staffing	191	2.84	0.706	173	3.26	0.759	0.000
Handoffs and transitions	197	3.27	0.613	171	3.25	0.619	0.977
Nonpunitive response to errors	195	2.43	0.616	170	2.59	0.591	0.006

Notes: *^)^ Based on Likert scale (1 = never/strongly disagree, 5 = always/strongly agree. Negatively worded questions have been recoded
